# A new genus and species of nudibranch-mimicking Syllidae (Annelida, Polychaeta)

**DOI:** 10.1038/s41598-024-66465-4

**Published:** 2024-07-29

**Authors:** Naoto Jimi, Temir A. Britayev, Misato Sako, Sau Pinn Woo, Daniel Martin

**Affiliations:** 1 Sugashima Marine Biological Laboratory, Graduate School of Science, Nagoya University, 429-63 Sugashima, Toba, Mie 517-0004 Japan; 2 Centre for Marine & Coastal Studies, Universiti Sains Malaysia, USM, 11800 Penang, Malaysia; 3 Laboratory of Marine Invertebrates Morphology and Ecology, A. N. Severtzov Institute of Ecology and Evolution, Russian Academy of Sciences, Moscow, Russian Federation; 4 Department of Marine Ecology, Centre d’Estudis Avançats de Blanes (CEAB-CSIC), Blanes, Catalunya Spain

**Keywords:** Annelida, Syllidae, Symbiosis, Polychaeta, Taxonomy, Biodiversity, Ecology, Evolution, Zoology

## Abstract

Nudibranch mollusks, which are well-known for their vivid warning coloration and effective defenses, are mimicked by diverse invertebrates to deter predation through both Müllerian and Batesian strategies. Despite extensive documentation across different taxa, mimickers have not been detected among annelids, including polychaetes, until now. This study described a new genus and species of polychaete living on *Dendronephthya* octocorals in Vietnam and Japan. Belonging to Syllidae, it exhibits unique morphological adaptations such as a low number of body segments, simple chaetae concealed within the parapodia and large and fusiform antennae and cirri. Moreover, these appendages are vividly colored, featuring an internal dark red area with numerous terminal white spots and bright yellow tips, effectively contributing to mimicking the appearance of a nudibranch. This discovery not only documents the first known instance of such mimicry among annelids, but also expands our understanding of evolutionary adaptation and ecological strategies in marine invertebrates.

## Introduction

Nudibranch mollusks are well-known for showcasing brilliant warning colorations advertising their efficient defenses, which allow them to move around seemingly with impunity facing potential predators^1,2^. This characteristic has fostered the evolution of mimics, either non-defended (Batesian) or defended (Müllerian)^3,4^. Although nudibranch mimicry has been documented in various taxa such as platyhelminths, arthropods, and echinoderms^1,5^, as well as among other nudibranchs^6^, it has never been found among annelids, including polychaetes. This paper unveils the existence of a stunning species of annelid polychaete from the family Syllidae closely resembling a nudibranch.

Syllidae is the largest polychaete family, including more than 1100 species across 79 genera with varying degrees of species diversity (Martin et al., 2021). The family encompasses five monophyletic subfamilies (Anoplosyllinae, Eusyllinae, Autolytinae, Exogoninae, and Syllinae), along with several taxa considered *incertae sedis*^7–11^. Syllids are distributed globally and exhibit high abundance and diversity across many marine ecosystems, ranging from the mesolittoral and shallow intertidal zones to the deep sea^12^. Their habitats span interstitial to cryptofaunal environments on both soft and hard substrates, their lengths vary from less than 1 mm to approximately 15 cm^13^, and their bodies show the typical linear polychaete organization^14^, with a few exceptions featuring branching bodies^15–17^. Species identification is often challenging for this family of polychaetes. Nevertheless, the family is generally recognizable due to the proventricle — a muscular, barrel-shaped pharyngeal structure whose pumping contributes to an omnivorous feeding habit, often facilitated by different types of terminal pharyngeal teeth^13,18,19^.

While most syllids adopt a free-living lifestyle, the known number of symbiotic species is rapidly expanding^20^. Serving as hosts for various organisms, including bacteria, ciliophorans, haplosporidians, and other polychaetes^21–24^, they primarily form symbiotic relationships with invertebrate hosts such as sponges, cnidarians, bryozoans, echinoderms, and tunicates^20,25–29^.

Many living syllids show vivid color patterns that are often crucial for species identification but tend to fade in preserved specimens^30–32^. In free-living species, these color patterns may play an aposematic role, while in symbionts they may function as camouflage. A renowned example occurs within *Alcyonosyllis* Glasby & Watson, 2001 (Syllinae), whose species are associated with anthozoans, including one scleractinian coral belonging to *Goniopora* de Blainville, 1830 and at least four malacalcyonacean octocorals belonging to *Xenia* Lamarck, 1816, *Melithaea* Milne Edwards, 1857 and *Dendronephthya* Kükenthal, 1905^20,33–36^.

**Figure 1:**
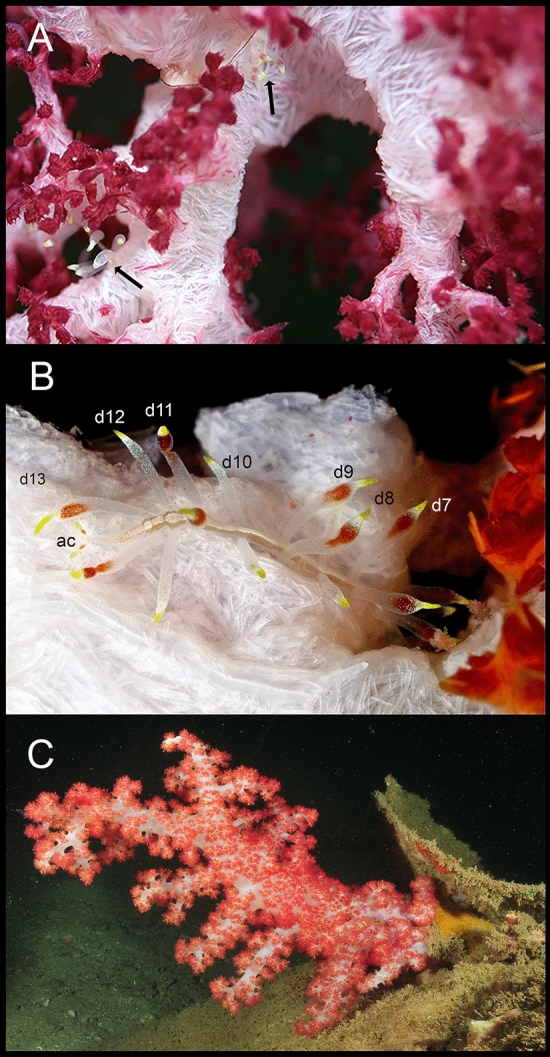
*Cryptochaetosyllis imitatio* gen. et sp. nov. on its hosts: (**A**). two Japanese specimens on *Dendronephthya* aff. *tenera* (black arrows). (**B**). Vietnamese specimen on *Dendronephthya* sp., with its anterior region hidden between the host branches; d1–d13: dorsal cirri; ac: anal cirri. (**C**). Specimen of *Dendronephthya* sp. from Vietnam. Photos in A by N. Ueda and in (**B**–**C**) by O. V. Savinkin.

We focused on *Dendronephthya* as a potential host, which prompted two independent field trips to explore the symbiotic fauna associated with species of this genus in Vietnam and Japan (Fig. 1A–C). During these surveys, we chanced upon a remarkable syllid thriving on species of this octocoral and boasting a significantly altered, brightly colored body that closely resembled a nudibranch (Fig. 1A, B). Through morphological and molecular analyses, we identified it as a new genus and species within the subfamily Autolytinae. In this paper, we provide a comprehensive description and illustrations of this newfound worm, offering insights into the significance of our discovery and the potential nature of its mimicry toward nudibranchs.

## Materials and methods

In Japan, specimens of the host species *Dendronephthya* sp. aff. *tenera* (Fig. 1A, B) were collected by SCUBA diving off Koza, Wakayama, and by gill net off Sugashima Island, Japan, on 19 May 2022 and 03 November 2021, respectively. Syllids were removed from the octocorals after anesthetizing with MgCl_2_, photographed, fixed in 70% ethanol and observed under stereomicroscopes (Nikon SMZ800N and Nikon). To preserve the integrity of the anterior end, neither the holotype, nor the paratype was dissected to observe the pharyngeal armature. However, a transverse section of a segment with long dorsal cirri of the epitokous region of the paratype was dissected and mounted on a slide before it was observed and photographed under a Nikon ECLIPSE Ni light microscope attached to a Nikon D6500 digital camera. The posterior end of the body, including several posterior segments of the atokous region and the anterior segments of the epitokous region of the paratype, was also cut off and post-fixed in 2% OsO4 for 2 h, dehydrated through a series of ethanol and acetone, critical point dried (BAL-TEC CPD-030), osmium coated (Filgen OPC40), and observed using JEOL JSM-7001F scanning electron microscope. The type specimens were deposited in the National Museum of Nature and Science (NSMT), Japan.

In Vietnam, the specimens of *Dendronephthya* sp. (Fig. 1C) were inspected for the presence of symbionts by SCUBA diving at 15–20 m depth at Mun Island, Bay of Nhatrang in October 2008. The soft coral and a single syllid were photographed in situ and collected. More photographs were taken in the laboratory before being preserved in 70% ethanol. Unfortunately, this specimen was lost at some time when the collections were moved to the new facilities of the Laboratory of Marine Invertebrates Ecology and Morphology in Moscow.

Total DNA of the Japanese specimen was extracted from a dorsal cirrus of the holotype using a DNeasy Tissue Kit (Qiagen). DNA extraction, sequencing, alignment, and removal of ambiguous positions were carried out according to Jimi, et al.^37^. The newly obtained sequences were deposited in GenBank (Reference numbers PP545372, PP545378, and PP545379). A total of 107 sequences (41 species) were used for molecular analyses. A total of 105 sequences were downloaded from GenBank see table 1 in^38^. The remaining sequences were obtained from this study. The phylogenetic analysis was also carried out according to Jimi, et al.^37^, with *Odontosyllis fulgurans* (Audouin & Milne Edwards, 1833) and *Odontosyllis gibba* Claparède, 1863 as outgroups.

The COI region sequences of *Epigamia macrophthalma* (Marenzeller, 1875) and our new species were used for calculating K2P genetic distances via MEGAX^39^.

The electronic edition of this article conforms to the requirements of the amended International Code of Zoological Nomenclature (International Commission on Zoological Nomenclature 2018), and hence the new names contained herein are available under that Code from the electronic edition of this article. This published work and the nomenclatural acts it contains have been registered in ZooBank, the online registration system for the ICZN. The ZooBank LSIDs (Life Science Identifiers) can be resolved and the associated information can be viewed through any standard web browser by appending the LSID to the prefix http://zoobank.org/. The LSID for this publication is: urn:lsid:zoobank.org:pub: FC92DF85-E808-438C-9292-5F4286D41D2B. The electronic edition of this work is published in a journal with an ISSN, and has been archived and is available from the following digital repositories: ResearchGate and DigitalCSIC.

## Results

### Molecular analysis

**Figure 2:**
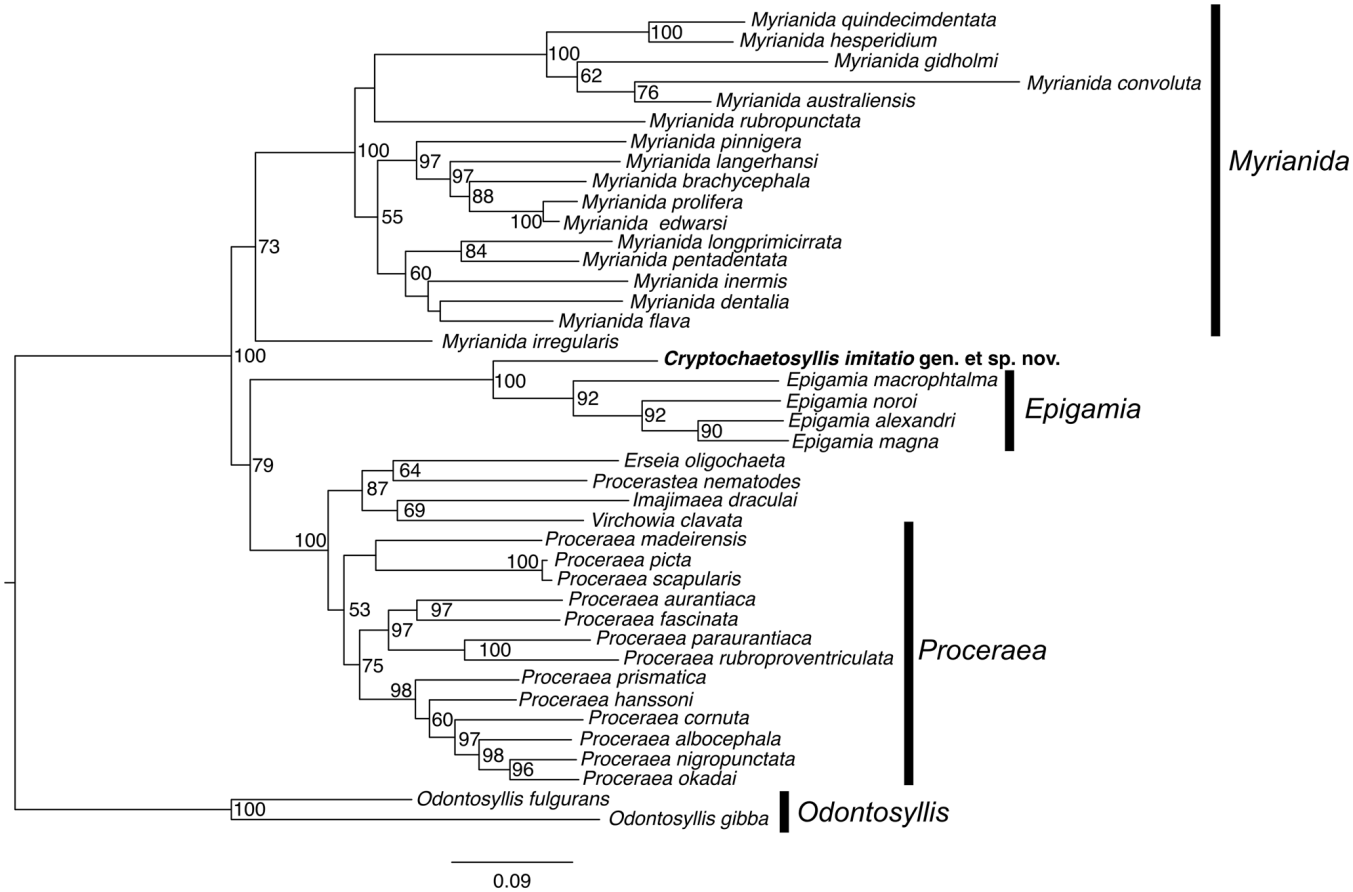
Phylogenetic tree (ML) showing the position of *Cryptochaetosyllis imitatio* gen. et sp. nov. as sister of the *Epigamia* clade.

Our phylogenetic tree (Fig. 2) grouped the sequences obtained from the Japanese specimen within the Autolytinae with a 100% bootstrap support [BS] and was a sister to *Epigamia* Nygren, 2004 with high support (100% BS). The clade formed by the Japanese sequence and *Epigamia* is a sister to *Proceraea*, with moderate support (79% BS).

The sequence obtained from the Japanese specimen differed by 24.7% (COI) and 26.1% (16S) from those of the most closely related species of *Epigamia* (*E*. *macrophthalma*), which are more than 10 times greater than the species discrimination threshold in polychaetes^40,41^, thus supporting the erection of the new genus.

### Systematics

***Cryptochaetosyllis*** Jimi, Britayev and Martin gen. nov.

[New Japanese name: keshou-syllis].

urn:lsid:zoobank.org:act:2A3D96BF-8829-4B3C-82EC-2D41638A3BC1.

#### Type species

*Cryptochaetosyllis imitatio* gen. et sp. nov.

#### Diagnosis

Autolytinae with short, cylindrical body, three antennae, palps absent and one pair of tentacular cirri. Parapodia of atokous adults with aciculae and simple hooked chaetae, all embedded inside chaetal lobe; notopodial natatory chaetae in epitokous region. Dorsal cirri alternating long, fusiform, similar in shape but much longer and wider than central antennae, with short, cylindrical, similar in shape to lateral antennae and tentacular cirri. Central antennae and tentacular and long dorsal cirri with brightly pigmented distal ends in living specimens. Ventral cirri absent in all parapodia. Reproduction by schizogamy (stolonization), with single stolons and marked sexual dimorphism (*Polybostrichus*, male, and *Sacconereis*, female).

#### Etymology

The new genus name, feminine in gender, derives from *Crypto* (covered in Latin) + *chaetae* + *syllis* (a Greek Sicyonian nymph), referring to the fact that chaetae in the atokous region of the single known species remain covered by tissue inside the chaetal lobe.

#### Remarks

The presence of the proventricle and the characteristic epitokous stolons lends support to the classification of the new genus within Syllidae, a conclusion reinforced by our phylogenetic analysis, which placed it within the Autolytinae. The simple chaetae concealed within the parapodia, coupled with the large fusiform dorsal cirri, are hallmark and unique features defining this genus.

***Cryptochaetosyllis imitatio*** Jimi, Britayev and Martin sp. nov.

[Japanese name: keshou-syllis].

(Figs. 1, 3–5).

**Figure 3:**
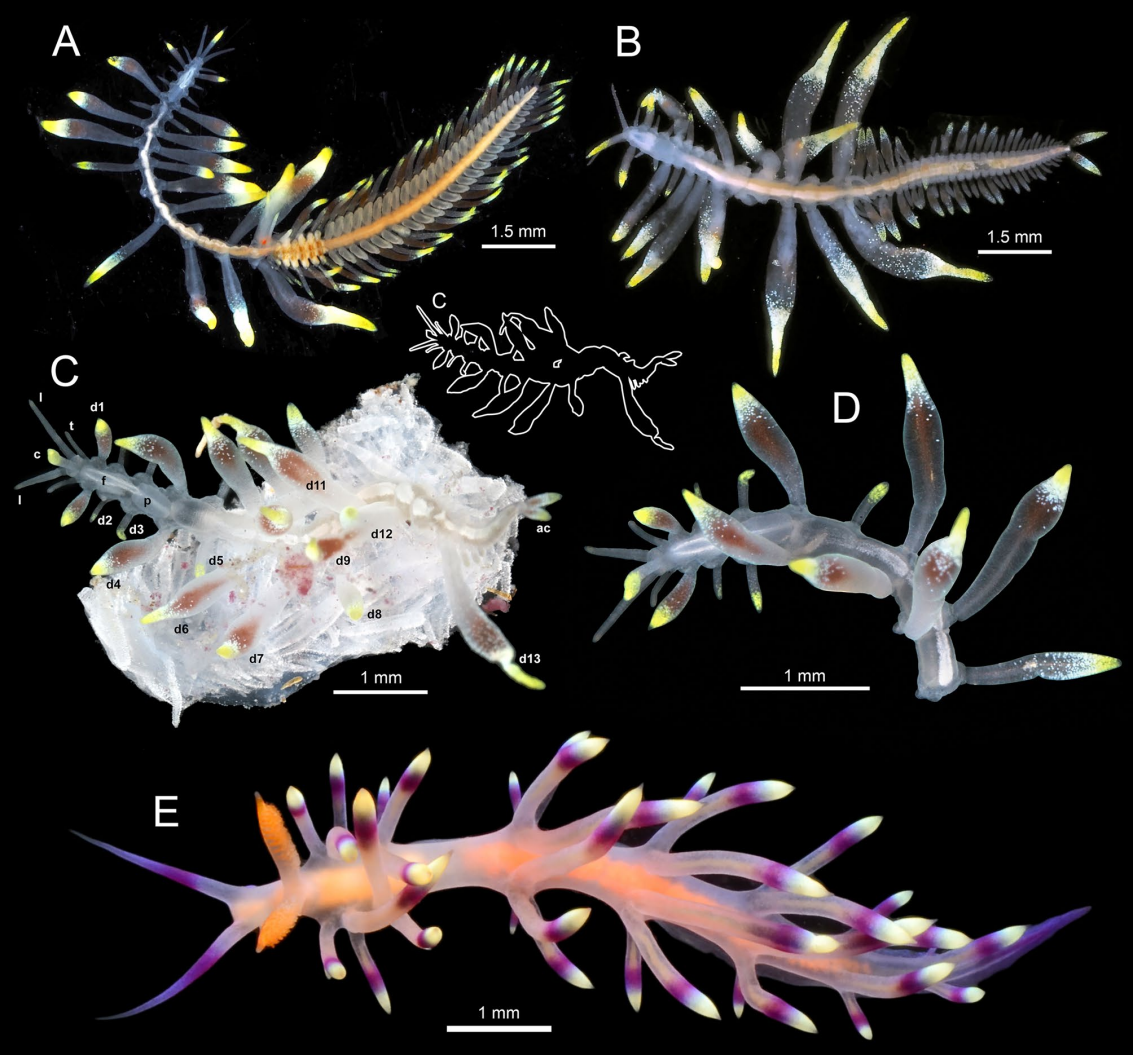
*Cryptochaetosyllis imitatio* gen. et sp. nov. Living specimens. (**A**). Holotype NSMT-Pol H-928, Japanese female with *Sacconereis* stolon. (**B**). Paratype NSMT-Pol P-929, Japanese male with *Polybostrichus* stolon. (**C**). Vietnamese specimen on *Dendronephthya* sp.; c: central antenna; l; lateral antennae; d1–d13: dorsal cirri; p, pharynx; ac: anal cirri; top right scheme: worm outline. (**D**). Vietnamese specimen, lacking posterior-most segments. (**E**). A specimen of *Coryphellina exoptata* from the Bay of Nhatrang (Vietnam). Photos in (**C**–**D**) by O. V. Savinkin and in E by T. I. Antokhina.

urn:lsid:zoobank.org:act: 69867538-F105-4616-9F1B-516C0622FC9C.

#### Diagnosis

Species of *Cryptochaetosyllis* with fourteen segments and thirteen dorsal cirri, regularly alternating long, thick and fusiform (L) with short, thin and cylindrical (S) as: L S, S, L, S, L, L, S, L, S, L, S, L.

#### Materials examined

Holotype (NSMT-Pol H-928), 6 mm long, 1 mm wide, Off Koza, Wakayama (33°29′57"N, 135°50′15"E), 45 m depth, on *Dendronephthya* aff. *tenera*, fixed in 70% ethanol, collected by N. Jimi. Paratype (NSMT-Pol P-929), 1 specimen, 5 mm long, 1 mm wide, Off Sugashima (34°29′40"N, 136°54′58"E), 40 m depth, on *Dendronephthya* aff. *tenera*, fixed in 70% ethanol, collected by N. Jimi. 1 additional specimen (anterior fragment, eight segments, specimen lost, documented by photos “in vivo”), Mun Island, Bay of Nathrang, Vietnam (12°9′47''N, 109° 18′10''E), at 15–20 m depth, on *Dendronephthya* sp., preserved in 70% ethanol, collected by O.V. Savinkin on October 27 2008.

#### Etymology

The specific epithet “*imitatio*” (mimicking in Latin) refers to the body shape and coloring of the species, which mimics sea slugs.

#### Description

Based on Japanese specimens (data on Vietnamese specimen between brackets). Body cylindrical, 5–6 mm long, 1 mm wide, with 14 segments in atokous anterior region and 31–33(23) segments in epitokous posterior region (Fig. 3A–D).

**Figure 4:**
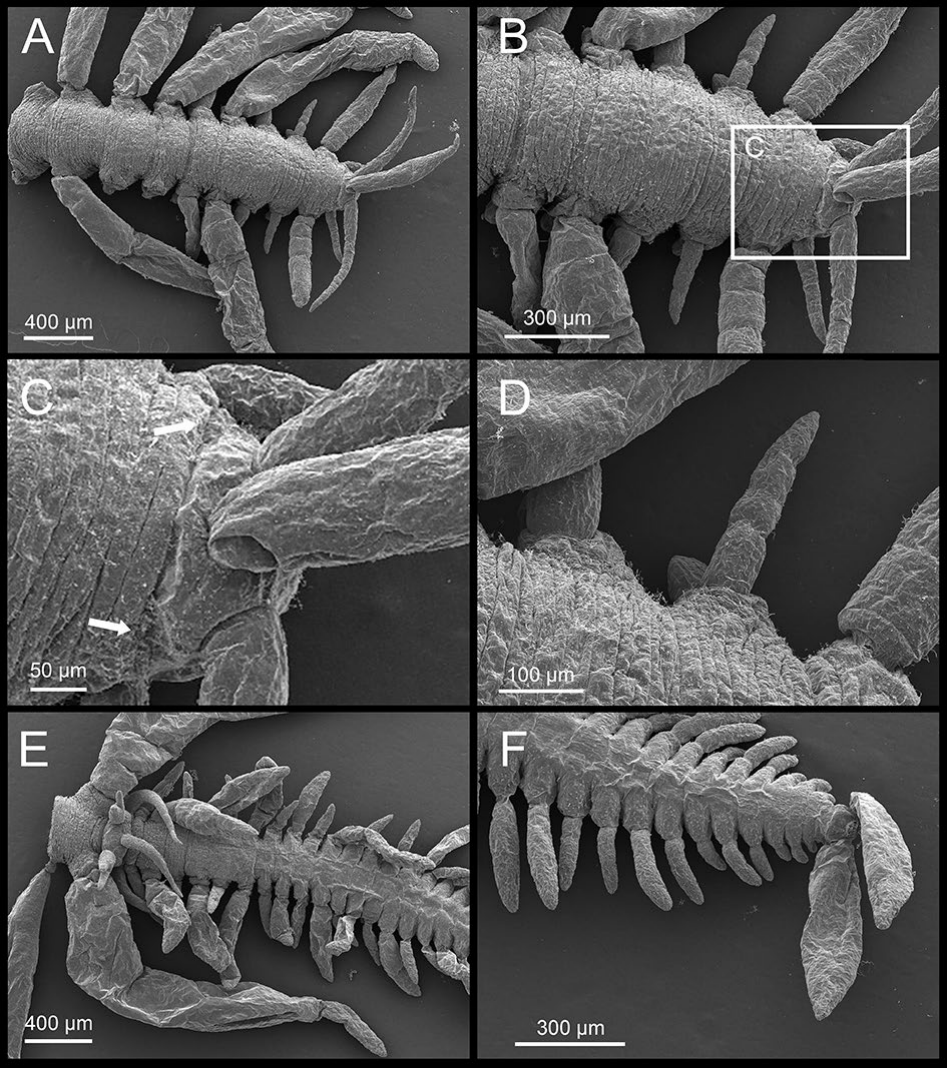
*Cryptochaetosyllis imitatio* gen. et sp. nov. SEM micrographs of Japanese male with *Polybostrichus* stolon, Paratype NSMT-Pol P-929, dosal view. A. Anterior region. B. Close view of anterior region shown in (**A**). (**C**). Close view of prostomium/peristomium in region shown in (**B**), with arrows pointing at ciliated nuchal organs. (**D**). Parapodia with short, wipe-shaped cirri. E. Posterior end of the atokous region and anterior end of the epitokous region. (**F**). Posterior end of the epitokous region.

**Figure 5:**
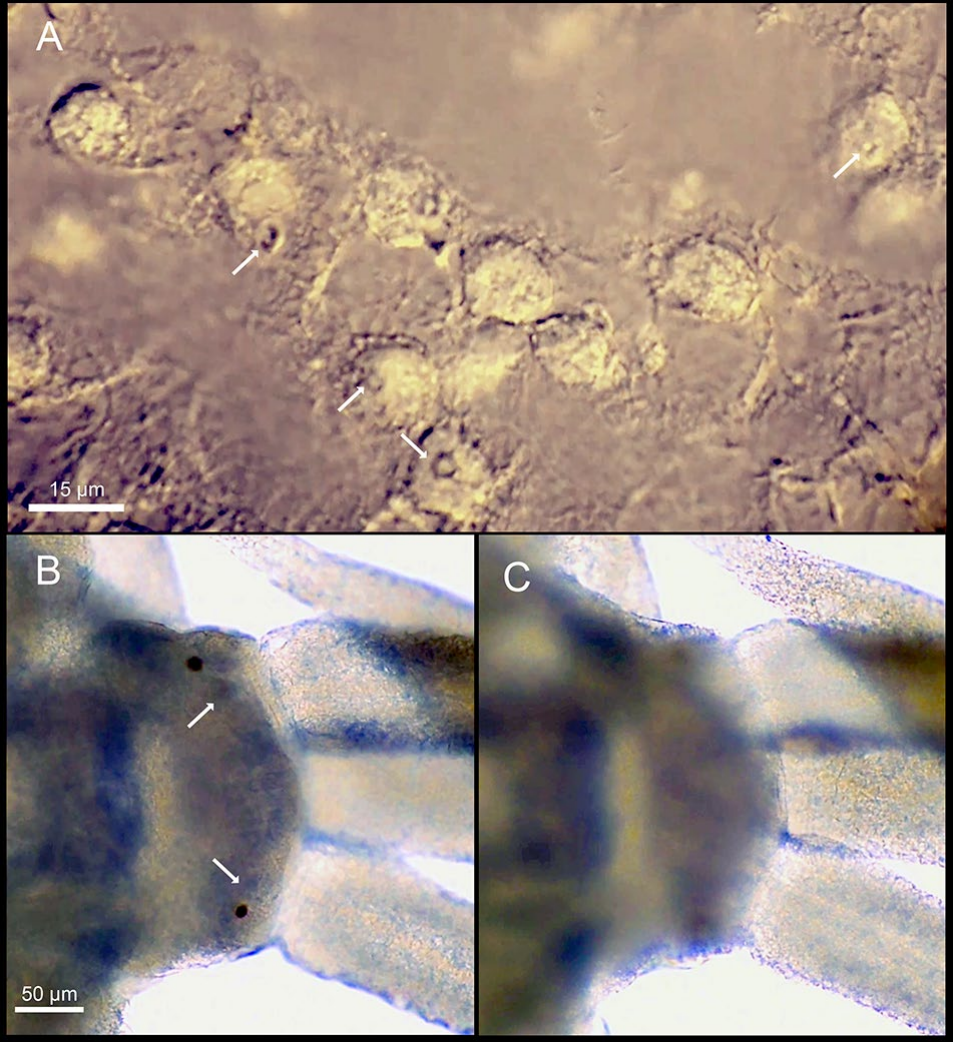
*Cryptochaetosyllis imitatio* gen. et sp. nov. Holotype NSMT-Pol H-928. (**A**). Glands from antennae and cirri; arrows pointing at gland openings. Light micrographs of the anterior end: (**B**). dorsal view; (**C**). ventral view; arrows pointing to the anterior pair of eyes.

**Figure 6:**
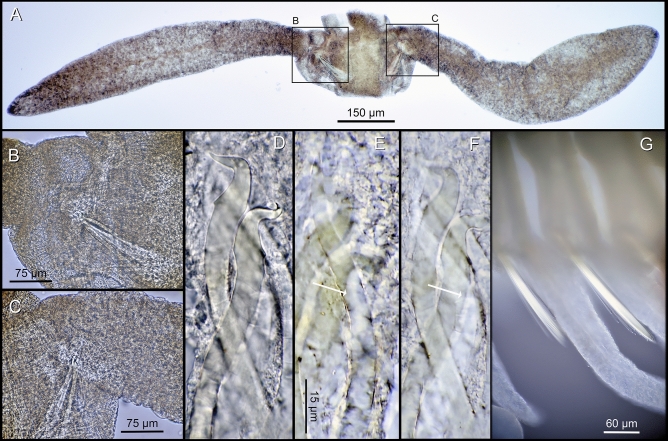
*Cryptochaetosyllis imitatio* gen. et sp. nov. Holotype NSMT-Pol H-928. Light micrographs. Atokous region. (**A**). Transverse section of a parapodia with long, fusiform dorsal cirri. (**B**). Details of the left parapodium. (**C**). Details of the right parapodium. (**D**). Simple, hooked, internal chaetae. (**E**–**F**). Sigmoid aciculae; arrows point to the middle of each acicula. Epitokous region. (**G**). Parapodia showing the natatory chaetae.

**Figure 7:**
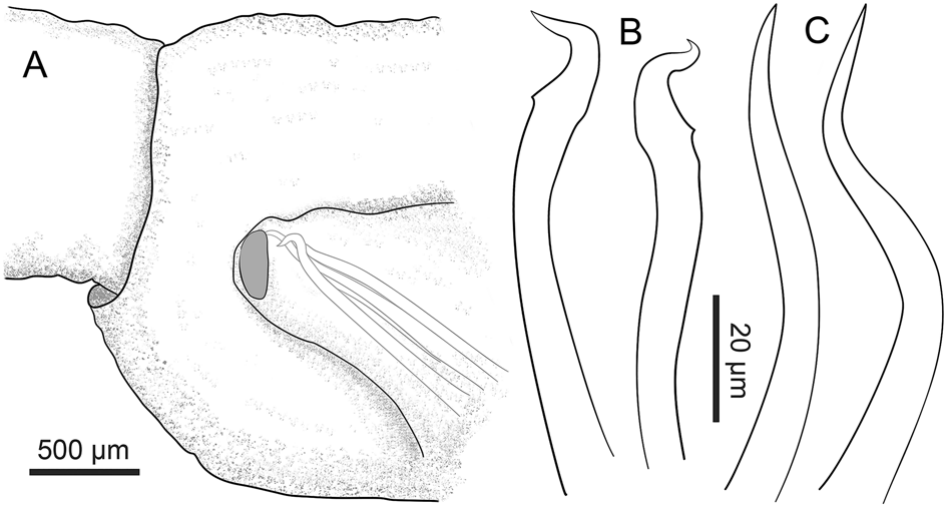
*Cryptochaetosyllis imitatio* gen. et sp. nov. Holotype NSMT-Pol H-928. (**A**). Scheme of a parapodia showing the chaetal lobe with the chaetae and aciculae embedded inside. (**B**). Simple hooked chaetae. (**C**). Sigmoid aciculae.

Atokous region with segmentation clearly visible dorsally and ventrally, particularly in preserved specimens (Fig. 4A–B). Living specimens transparent, with dark red eyes; antennae pigmented in white and yellow at distal end, more evident in central antennae; dorsal cirri pigmented, with internal dark red area, numerous terminal white spots and bright yellow tips, more evident in long cirri (Fig. 3A–D). White spots corresponding to roughly spheric glands, ca. 15–20 µm in diameter, with minute circular pore openings and indistinct granular contents (Fig. 5A, Video 1). Prostomium oval, with four eyes arranged in a rectangle, anterior pair below basis of lateral antennae; palps absent; median antenna inserted dorsally on middle of prostomium, smooth, fusiform, as long as, or slightly shorter than, lateral antennae, with central, enlarged region twice thick than in lateral antennae; two lateral antennae inserted frontally at anterior end of prostomium, smooth, cylindrical, gradually tapering (Figs. 3A–D, 4A, B, 5B, C, Video 2). Nuchal organs as ciliated pouches between prostomium and peristomium (Fig. 4A–C). Pharyngeal opening, tooth and/or trepan not seen; pharynx straight (i.e., non-convoluted), 1.5 times longer and ca. four times thinner (three times) than proventricle, extending for about 2.5 segments; proventricle rectangular, ca. 1.5 times longer than wider, occupying 1.5–2 segments (Fig. 3A–C). One pair of tentacular cirri, smooth, similar in shape but twice shorter than lateral antennae (Figs. 3A–C, 4A, B). All dorsal cirri smooth; first pair of 1/3 longer than tentacular cirri, fusiform, similar in shape to central antennae; second and third pairs short, cylindrical with gradually pointed end, similar in shape (but 1/3 shorter than and similar in length, respectively) to tentacular cirri; fourth pair long, fusiform, similar in shape to first pair, ca. twice longer and thicker; fifth pair short, cylindrical, similar to third pair, but 1.5 times longer and thicker; sixth and seven pairs long, fusiform, similar in shape, length and width to fourth pair; eight pair cylindrical, slightly shorter and thinner than seven; from eighth to thirteenth pairs, cirri regularly alternating long, thick and fusiform with short, thin and cylindrical, gradually increasing in length, with thirteen pair being longest (Figs. 3A–D, 4A). From segment 3, parapodia as single small globular projections or lobes, with a brownish oval spot at tip, partly covering distal ends of chaetae and aciculae; ventral cirri absent (Figs. 4B, D, 6A, 7A). Parapodia with two simple, unidentate, hooked chaetae, each with a small subdistal boss and a fine, upwards recurved tip, and two sigmoid aciculae with pointed tips; chaetae and acicula internal, non-protruding outside external surface of chaetal lobe (Figs. 6A–F, 7A–C, Video 3). Two anal cirri, seen only in epitokous regions (Figs. 3A–C, 4F).

Holotype female epitokous region of *Sacconereis*-type (Fig. 3A), attached to segment 14 of atokous region, with 33 segments. Four large (anterior pair larger than posterior) red eyes. Antennae with same color pattern as those in atokous region, three, wipe-shaped, thinner and slightly short than following dorsal cirri. First six segments with pale orange parapodia and brownish segmental bands dorsally; from segment 7, all parapodia transparent, globular, ca. twice as long as parapodia of anterior-most segments, with a central, longitudinal orange band present until most posterior segments. Dorsal cirri with pigmentation similar to those of atokous region, all of about same length, irregularly alternating between relatively short, slightly wipe-shaped (with white and yellow distal ends and red inner color almost absent, S) and longer, slightly fusiform (with dark red inner color and white and yellow distal ends, L), as S, L, S, L, S, L, S, L, S, L, S, L, S, L, S, S, L, L, S, S, L, L, S, S, L, L, S, L, L, S, L, S, S. Ventral cirri absent. Anterior segments with natatory notochaetae, simple, blade shaped, with tapering tips. Two anal cirri, similar in shape and coloring to more fusiform dorsal cirri.

Paratype male epitokous region of *Polybostrichus*-type (Figs. 3B, 4E, F), attached to segment 14 of atokous region, with 31 segments. Four eyes large similar in size, blackish. Two bifurcated palps, transparent. Three antennae, wipe-shaped, 1.5 times longer than dorsal cirri of first segment, with whitish and yellowish coloring at tips poorly developed. All segments along body similar in shape, with short, globular, transparent parapodia. First segment achaetous, with dorsal cirri wipe-shaped, thinner and half shorter than following dorsal cirri; second segment with fusiform dorsal cirri and pigmentation similar to those of atokous region, then irregularly alternating long, fusiform well-pigmented (L) with short, wipe shaped, less pigmented (S) till body end, as S, L, S, L, S, L, S, L, S, L, S, L, S, L, S, S, L, L, S, S, L, L, S, S, L, L, S, L, L, S, L, S. Ventral cirri absent. Anterior segments with natatory notochaetae, simple, blade shaped, with tapering tips (Fig. 6G). Two anal cirri, similar in shape and coloring to anterior-most fusiform dorsal cirri.

#### Variation

The Japanese specimens comprised a female and a male exhibiting fully developed *Sacconereis* and *Polybostrichus* stolons attached to their posterior ends. In contrast, the Vietnamese specimen displayed 23 (instead of 31–33) segments, not well-defined and having with minute and nearly colorless dorsal cirri (Fig. 3C), while the natatory notochaetae and colorful anal cirri were identical to those of the mature Japanese stolons. This strongly corroborates its posterior region as being an early-stage epitokous, rather than merely regenerating segments post stolon release. Furthermore, the Vietnamese specimen differed from their Japanese conspecifics by featuring a slightly more pronounced dark red color in the enlarged central section of the fusiform dorsal and annal cirri.

#### Distribution and ecology

Known from Koza, Wakayama (type locality) and Sugashima in Japan and from Mun Island (Bay of Nathrang) in Vietnam, from 10–45 m depth, found living in association with the soft coral *Dendronephthya* aff. *tenera* in Japan (Fig. 1A, B) and *Dendronephthya* sp. in Vietnam (Fig. 1C). The species seems to be very rare, as we have been able to find four specimens in Japan, two only photographed (Fig. 1A) and two collected (Fig. 3A, B) and only one in Vietnam, collected but later lost (Figs. 3C, D), after checking hundreds of potential host octocorals. On the other hand, the prevalence of the infestation can be 1–2, as the two only photographed Japanese specimens shared the same host.

## Discussion

Nudibranchs are renowned for their aposematic coloration and for having numerous examples of Batesian and Mullerian mimickers, not only among other invertebrates but also within their own taxa^1,5,6^. However, until now, there has been no documented case of nudibranch mimicry involving annelids. Remarkably, no instances of Mullerian mimicry have been reported thus far among annelids. Conversely, Batesian mimicry has been tentatively suggested for some species, although with limited documentation^42^. An intriguing example is the freshwater leech *Macrobdella diplotertia* Meyer, 1975, which appears to mimic the swimming behavior and coloration of the noxious newt *Notophthalmus viridescens louisianensis* (Wolterstorff, 1914)^43^. In this way, the mimicking leech gains protection and, since the newt poses no threat to fish, it may go unnoticed, facilitating a close approach and enhancing predatory opportunities.

In marine environments, holopelagic swimming polychaetes of the genera *Drieschia* (Polynoidae) and *Tomopteris* (Tomopteridae) exhibit Batesian behavioral mimicry. These elongated worms can remain motionless when disturbed, curling their bodies to resemble unpalatable or well-defended medusae^42,44,45^. The elongated cirri of *Drieschia*, believed to enhance swimming ability, may also contribute to their resemblance to medusae when curled^42^. For *Tomopteris*, alternative hypotheses for body curling unrelated to Batesian mimicry, include the potential to deter predation by increasing body diameter and producing mucus and to prevent physical damage when in contact with other organisms^45^.

The aforementioned marine genera are holoplanktonic and free-living, and with the exception of the elongated tentacles in *Drieschia*, their purported Batesian mimicries result from behavioral adaptations. In contrast, the newly discovered, *C. imitatio* gen. et sp. nov. is a benthic species consistently found on *Dendronepthtya* that has simple hooked chaetae and is common in many symbiotic polychaetes, including the other syllids such as the species of *Haplosyllis* and *Alcyonosyllis*^20,46^, and the morphology and color of its dorsal cirri mimic coral polyps, a trait also typical of symbionts. Nevertheless, its overall body coloration may function aposematically depending on circumstances. Another autolytinid syllid, *Proceraea janetae* Martin, Gil, Abgarian, Evans, Turner & Nygren, 2015, displays vivid aposematic coloration and regularly visits the scleractinian coral *Montastraea cavernosa* (Linnaeus, 1767) to dig with its anterior end inside the gastral cavity of the coral polyps, being either a kleptoparasite (i.e., stealing food) or a specialized predator (i.e., feeding on polyp tissues)^30^. Consequently, additional observations are crucial to discern the true nature of the association of *C. imitatio* gen. et sp. nov. with the octocoral, as it may be a symbiont but also a specialized predator recurrently visiting the alcyonacean for feeding. In both scenarios, it could capitalize on mimicry (when on the coral) and aposematic coloration (when moving outside the coral).

On the other hand, the body color and morphology of *C. imitatio* gen. et sp. nov. closely mimic those of a nudibranch, marking the first documented instance of sea slug mimicry within Annelida. While our observations cannot confirm whether *C. imitatio* gen. et sp. nov. simulates nudibranch behavior, we can assume that there are likely no significant discrepancies in the way both types of animals move. Notably, its mimicry is enhanced by the fact that the hooked chaetae remaining concealed within the parapodia. Hooked simple chaetae, a common feature of symbiotic polychaetes, are interpreted as simplified compared to their more complex counterparts in free-living species^46^. In syllids, this simplification often arises through the fusion of blades and shafts of their typical articulated chaetae^47,48^, which may also apply to *C. imitatio* gen. et sp. nov. Specifically, the chaetae of our specimens closely resemble those found in the anthozoan-associated species of *Alcyonosyllis*^33–36^ or in the gorgonian associated *Haplosyllis chamaeleon* Laubier, 1960 and *Haplosyllis villogorgicola* Martin, Núñez, Riera & Gil, 2002^47^. Both *Alcyonosyllis* and *Haplosyllis* are Syllinae but appear to be phylogenetically unrelated^49^, while *C. imitatio* gen. et sp. nov. is an Autolytinae. Thus, we may hypothesize that similar chaetal morphologies have independently evolved as a convergent adaptation to facilitating attachment or movement on their host anthozoans. Accordingly, we cannot rule out the possibility that the hidden chaetae of *C. imitatio* gen. et sp. nov. could be retractable, thus covering a dual influence: attachment and movement on the host (when protruded), as well as facilitating nudibranch mimicry (when retracted).

Indeed, *C. imitatio* gen. et sp. nov. exhibits striking similarities to certain nudibranch species reported from the same regions where the worm is found, such as *Samla bicolor* (Kelaart, 1858), *Samla takashigei* Korshunova, Martynov, Bakken, Evertsen, Fletcher, Mudianta, Saito, Lundin, Schrödl & Picton, 2017, or *Coryphellina exoptata* (Gosliner & Willan, 1991), all them belonging to the family Flabellinidae^50–53^. *Samla bicolor* have numerous reports in the Indo-West Pacific and, in fact is a species complex, among which *S. takashigei* has been recently described as new species^50^. The species of the complex are characterized by narrow body with whitish background, small number of long, fusiform cerata with yellowish to brownish subdistal parts and apical tips with or without a cap of white pigment, depending on the different color morphotypes^50–53^. Other species of *Samla* have similar coloring, but their geographical distribution is very far from our study site^53^. Another common species of nudibranch in Nhatrang Bay, *Coryphellina exoptata*, tends to be more pinky than whitish; however it resembles *C. imitatio* gen. et sp. nov. in having a narrow, small body and a reduced number of fusiform cerata showing subdistal reddish bands and whitish-yellowish tips (Fig. 3E)^50,53^.

While most aeolid nudibranchs feeding on hydrozoans possess cnidosacs (specialized organs containing functional hydrozoan cnidocytes) in their cerata for defense against predation^54^, our observations do not confirm whether *C. imitatio* gen. et sp. nov. has any defense mechanism other than its color and body shape. The dorsal cirri of the worm feature roughly spherical glands with minute openings and indistinct granular contents scattered inconsistently in the dermal tissue. As they lack any internal structure, we may discard them because they are sequestered cnidocytes.

Dorsal cirri glands are common in many other syllids, such as in *Syllis gracilis* Grube, 1840 Fig. 8E in^55^, but particularly in small interstitial species where they appear to enhance adherence to sand grains^56^. Glands are also present in the ramified symbiotic species *Ramisyllis multicaudata* Glasby, Schroeder & Aguado, 2012, where they produce a whitish substance that turns red upon extraction of a body fragment, signaling the onset of a degeneration process^57^. However, the exact function of these glans remains unknown, as in most syllids^56^.

The dorsal cirri glands of *C. imitatio* gen. et sp. nov. are also whitish in living specimens. However, we did not observe them secreting any product or changing in color during our manipulative observations (except for fading of white after fixation). Thus, their true function also remains obscure. This ambiguity holds for our final considerations: if the glands store or produce toxic or deterrent substances, regardless of whether they are sequestered from the coral, then *C. imitatio* gen. et sp. nov. would be a Müllerian mimic, marking the first known case within the entire Annelida. As there is no other apparent defense mechanism, if the glands do not play a defensive role, then *C. imitatio* gen. et sp. nov. would be a Batesian mimic. This would represent not only the first known case in Syllidae and among marine benthic symbiotic Annelida but also the fourth for the entire phylum, following the freshwater leech and the holoplanktonic *Tomopteris* and *Drieschia*.

*Cryptochaetosyllis imitatio* gen. et sp. nov. is not abundant or frequently encountered, at least at our study sites, although it seems to be more abundant in Japan (with four specimens) than in Vietnam (with only one specimen). However, finding new specimens is crucial for assessing the actual species distribution range, as well as the relationships between the Japanese and Vietnamese populations. Additionally, further observations and experiments with living specimens are needed to determine the type of relationship with *Dendronephthya*, the functional role of the concealed hooked chaetae, the possible presence of secondary metabolites, and ultimately, the nature of the mimicry exhibited by this new genus and species of autolytinid syllid.

## Data availability

Sequence data that support the findings of this study have been deposited in the GenBank (https://www.ncbi.nlm.nih.gov/genbank) with the primary accession code PP545372, PP545378, and PP545379.

### Supplementary Information

Supplementary Video 1. *Cryptochaetosyllis imitatio* gen. et sp. nov. Light microscopy images of the spheric glands of dorsal cirri, showing the minute circular pore openings. https://saco.csic.es/index.php/s/4GMWbxGEw4xKzcA

Supplementary Video 2. *Cryptochaetosyllis imitatio* gen. et sp. nov. Anterior end, showing the insertion of antennae and the absence of palps. https://saco.csic.es/index.php/s/tWze7Zx3ZaSsm7y

Supplementary Video 3. *Cryptochaetosyllis imitatio* gen. et sp. nov. Chaetae end aciculae embedded inside the parapodial lobe. https://saco.csic.es/index.php/s/SMi8ML4mxqR2Skr
